# Acceptability of intrapartum HIV counselling and testing in Cameroon

**DOI:** 10.1186/1471-2393-9-9

**Published:** 2009-02-27

**Authors:** Eugene J Kongnyuy, Enow R Mbu, Francois X Mbopi-Keou, Nelson Fomulu, Philip N Nana, Pierre M Tebeu, Rebecca N Tonye, Robert JI Leke

**Affiliations:** 1 Child and Reproductive Health Group, Liverpool School of Tropical Medicine, UK; 2 Department of Obstetrics and Gynaecology, Faculty of Medicine and Biomedical Sciences, University of Yaounde I, Yaounde, Cameroon; 3 Department of Microbiology and Infectious Diseases, Faculty of Medicine and Biomedical Sciences, University of Yaounde I, Yaounde, Cameroon

## Abstract

**Background:**

To assess the acceptability of intrapartum HIV testing and determine the prevalence of HIV among labouring women with unknown HIV status in Cameroon.

**Method:**

The study was conducted in four hospitals (two referral and two districts hospitals) in Cameroon. Labouring women with unknown HIV status were counselled and those who accepted were tested for HIV.

**Results:**

A total of 2413 women were counselled and 2130 (88.3%) accepted to be tested for HIV. Of the 2130 women tested, 214 (10.1%) were HIV positive. Acceptability of HIV testing during labour was negatively associated with maternal age, parity and number of antenatal visits, but positively associated with level of education. HIV sero-status was positively associated with maternal age, parity, number of antenatal visits and level education.

**Conclusion:**

Acceptability of intrapartum HIV testing is high and the prevalence of HIV is also high among women with unknown HIV sero-status in Cameroon. We recommend an opt-out approach (where women are informed that HIV testing will be routine during labour if HIV status is unknown but each person may decline to be tested) for Cameroon and countries with similar social profiles.

## Background

About 3.2 million infants and young children worldwide are infected with Human Immunodeficiency Virus (HIV) and more than 95% of them get the virus from their mother, usually during the intrapartum period [1,2]. The traditional method for preventing mother-to-child transmission (MTCT) of HIV is voluntary testing during antenatal care and provision of antiretroviral prophylaxis to the HIV-infected women and their newborns [3,4].

The World Health Organisation estimates that in developing countries, 32% of pregnant women give birth with no previous antenatal care [5]. Although universal antenatal HIV testing is standard in the USA, an estimated 40% of HIV transmissions in 2000 occurred among mothers whose HIV infection was unrecognized [6]. In settings where many women give birth with unknown HIV status either because of low antenatal attendance or otherwise, HIV testing during labour provides the last window of opportunity before delivery for interventions to reduce MTCT of HIV [7,8].

A recent study in the USA reported that out of 190 labouring women who underwent rapid HIV testing, 7 were found positive. On subsequent testing of the seven HIV positive women by Western Blot, 3 were confirmed positive, 2 were found negative and the results of 2 remained indeterminate [6]. In this study the sensitivity of rapid HIV testing was 100%, specificity 97.9%, positive predictive value (PPV) 42.9%, and negative predictive value 100%. The authors suggested that availability of rapid HIV testing on labour and delivery units should not replace rigorous prenatal screening, which is currently the standard method screening for HIV during pregnancy. A two-step rapid HIV testing, particularly parallel testing, has been reported to be superior to single test strategy and is comparable to standard testing [4].

Acceptability of rapid intrapartum HIV testing varies from 83% to 97% depending on the study setting [4]. Homsy and colleagues in 2006 reported that 86% of labouring women in Tanzania accepted intrapartum HIV testing [9]. Malonza and colleagues (2003) compared rapid HIV testing and conventional HIV testing during antenatal care with respect to the percentage women who received their HIV test results in Kenya [10]. They found that 96% of women who had rapid HIV testing received their results compared to 73% of women who had conventional HIV testing.

Cameroon is one of the sub-Saharan African countries with high HIV prevalence (6.8% among women of reproductive age) [11]. In Cameroon, one in every five pregnant women do not receive antenatal care during pregnancy, meaning that traditional HIV testing during antenatal care cannot capture a sizeable proportion of women [11]. Of all women who attend antenatal care 92% accept HIV testing [12]. The objective of this study was to assess the acceptability of HIV testing among women who come in labour with unknown HIV status. This is the first study to evaluate the acceptability of intrapartum HIV counselling and testing in Cameroon.

## Methods

The study was conducted in two referral hospitals (Yaounde University Hospital Centre and Yaounde Central Maternity) and two district hospitals (Biyem-Assi and Cite Verte) between January 2004 and December 2005. All the four hospitals are located in the capital city, Yaounde. Both referral and district hospitals were chosen for this study in order to have a mix of labouring women who were representative of the capital city. The study was approved by the Ethics Committee of the Faculty of Medicine and Biomedical Sciences (University of Yaounde I).

All labouring women (except those in the second stage of labour) with unknown HIV status presenting to the labour room during the study period were eligible for inclusion in the study. No woman with unknown HIV status arrived in the second stage of labour. HIV counseling and testing was offered by a trained counselor and a laboratory technician round the clock. Counselors were midwives trained on appropriate counseling techniques and laboratory technicians were technicians who normally work in the hospitals; so there was no extra cost of recruiting new staff. Before the study started counselors were briefed about the study but did not receive any additional training. The basic elements of counseling were respected namely that clients understand: (i) the clinical and prevention benefits of testing, (ii) the right to refuse, (iii) the follow up services that will be offered, and (iv) the importance of sharing results with a partner in case of positive results. HIV positive women receive standard Nevirapine mono-therapy prophylaxis regimen immediately and their babies receive Nevirapine syrup.

We used rapid tests to screen for HIV. HIV was screened initially with Determine (Abbott Laboratories, IL, USA) and with Capillus (Trinity Biotec Plc, Wicklow, Ireland). When the results of the two tests were inconsistent, Unigold (Trinity Biotec Plc, Wicklow, Ireland) was used as tie-breaker. The results were disclosed to the patients within the shortest convenient time during a post-test counseling. Women tested HIV positive received psychological and social support through the labour from the counselor. Screens were used to ensure privacy and confidentiality.

Data was entered into SPSS Version 13. Descriptive statistics such as proportions were analysed and presented. Univariate analyses, using unadjusted odds ratios (OR) and Chi-square test for association, were conducted to assess the association between variables. Finally, multiple logistic regression analyses were then carried out and the results reported as adjusted ORs. Independent variables entered into the logistic models were age, parity, number of antenatal visits, marital status and level of education. All significance tests were two-tailed and statistical significance was defined at the 5% alpha level.

## Results

A total of 8043 pregnant women (2803 for Yaounde Central Hospital, 2510 for Yaounde University Hospital Centre, 1423 for Biyem-Assi District Hospital and 1307 for Cite Verte District Hospital) gave birth during the study period and 2413 (30.0%) of them did not have a HIV test done during pregnancy. All the 2413 women were counseled and 2130 (88.3%) accepted to be tested for HIV. Of the 2130 tested for HIV, 214 (10.05%) were HIV positive.

### Socio-demographic characteristics of study participants

Socio-demographic characteristics of the study participants are shown in Table 1. The mean age was 27 years (standard deviation [SD] = 8.5; range 15 to 45). A majority (57%) of the women were aged 20–29 years. Twenty one percent (21%) were nulliparous women, while 37.5% had four previous deliveries or more. More than 80% of women had at least one antenatal visit during their index pregnancy. Most of them were married (58%) and had secondary education or higher (57.2%).

**Table 1:** Socio-demographic characteristics of study population

***Characteristic***	***Number***	***Percentage (%)*** ***(N = 2413)***
**Age**		
< 20 years	493	20.4
20–29 years	1375	57.0
≥30 years	545	22.6
**Parity**		
0	507	21.0
1–3	1001	41.5
≥4	905	37.5
**No of antenatal visits**		
0	471	19.5
1–2	676	28.0
≥3	1266	52.5
**Marital status**		
Single	1013	42.0
Married	1400	58.0
**Education**		
No formal education	249	10.3
Primary	787	32.5
Secondary or higher	1380	57.2

### Acceptability of HIV testing

The associations between selected characteristics and acceptability of HIV testing of the study participants are presented in Table 2. Compared to younger women (< 20 years), women aged 20–29 years were 1.5 times (odds ratios [OR] = 0.68, confidence interval [CI] 0.46–0.97) and women aged ≥30 years 2 times (OR = 0.47, CI 0.34–0.73) more likely to refuse HIV testing in the labour room. Compared to nulliparous women, parous women were 1.6 times (OR = 0.63, CI 0.44–0.89) more likely to refuse HIV testing after counseling. Women who did not receive any antenatal care during their pregnancy were more likely to accept HIV testing than those who attended antenatal clinics 1–2 times (OR = 0.42, CI 0.26–0.66) or ≥3 times (OR = 0.49, CI 0.33–0.72). Compared to women with no formal education, women with primary education were 1.2 times (OR = 1.20, CI 0.81–1.86) and women with secondary education or higher were 1.7 times (OR = 1.65, CI 1.10–2.42) more likely to accept HIV testing after counseling. There was no significant association between acceptability of HIV testing and marital status

**Table 2:** Characteristics of women counselled in labour room by acceptability of HIV testing.

***Characteristic***	***Accepted HIV testing*** ***(N = 2130)*** ***(%)***	***Refused HIV testing*** ***(N = 283)*** ***(%)***	***Unadjusted OR (95% CI)***	***Adjusted OR (95% CI)***	***p-value***
**Age**					
< 20 years	445 (20.9)	38 (13.4)	1	1	
20–29 years	1214 (57.0)	161(56.9)	0.66 (0.45–0.95)	0.68 (0.46–0.97)	0.026
≥30 years	461 (21.6)	84(29.7)	0.46 (0.30–0.68)	0.47 (0.34–0.73)	< 0.001
**Parity**					
0	467(29.9)	40(14.1)	1	1	
1–3	874(41.0)	127(44.9)	0.59 (0.40–0.85)	0.63 (0.44–0.89)	0.005
≥4	789(37.0)	116(41.0)	0.58 (0.40–0.85)	0.60 (0.34–0.73)	0.004
**No of antenatal visits**					
0	441(20.7)	30(10.6)	1	1	
1–2	582(27.3)	94(33.2)	0.42 (0.27–0.64)	0.42 (0.26–0.66)	< 0.001
≥3	1107(52.0)	159(56.2)	0.47 (0.31–0.70)	0.49 (0.33–0.72)	< 0.001
**Marital status**					
Single	894(42.0)	119(42.0)	1	1	
Married	1236(58.0)	164(58.0)	1.00 (0.78–1.29)	1.00 (0.75–1.26)	0.980
**Education**					
No formal education	209(9.8)	40(14.1)	1	1	
Primary	682(32.0)	102(36.0)	1.28 (0.85–1.90)	1.20 (0.81–1.86)	0.225
Secondary or higher	1239(58.2)	141(49.8)	1.68 (1.14–2.45)	1.65 (1.10–2.42)	0.010

Overall, acceptability of HIV testing in the labour room was negatively associated with maternal age, parity and number of antenatal visits, but positively associated with level of education. The main reasons for refusal of rapid HIV testing were fear of stigma (35.3%), discrimination (29.3%), rejection (15.9%) and no reason specific reason or reason reserved (19.4%).

### Prevalence of HIV

Of the 2130 women tested for HIV, 214 were HIV positive, giving an overall HIV prevalence of 10.1% among labouring women with unknown HIV status.

The associations between selected characteristics and HIV status are presented in Table 3. Compared to younger women (< 20 years), older women (≥30 years) were more likely to be HIV positive (*p *= 0.035). Parous women also more likely to be HIV positive when compared to nulliparous women. Women who did not receive any antenatal care during their pregnancy were less likely to be HIV positive. Compared to women with no formal education, women with secondary education were more likely to be HIV positive (*p *= 0.006).

**Table 3:** Prevalence of HIV among labouring women with unknown HIV status by selected characteristics

**Characteristic**	**HIV positive** **(N = 214)** **(%)**	**HIV negative** **(N = 1916)** **(%)**	**Unadjusted OR** **(95% CI)**	**Adjusted OR (95% CI)**	**p-value**
**Age**					
< 20 years	36(16.8)	419(21.9)	1	1	
20–29 years	127(59.3)	1087(56.7)	1.36 (0.93–2.02)	1.33 (0.90–2.00)	0.127
≥30 years	56(26.2)	405(21.1)	1.61 (1.04–2.52)	1.58 (1.02–2.50)	0.032
**Parity**					
0	37(17.3)	430(22.4)	1	1	
1–3	103(48.1)	771(40.2)	1.55 (1.05–2.32)	1.51 (1.03–2.29)	0.028
≥4	74(34.6)	715(37.3)	1.20 (0.80–1.83)	1.10 (0.79–1.80)	0.383
**No of antenatal visits**					
0	29(13.6)	412(21.5)	1	1	
1–2	64(29.9)	518(27.0)	1.76 (1.12–2.80)	1.71 (1.09–2.75)	0.013
≥3	121(56.5)	986(51.5)	1.74 (1.15–2.69)	1.70 (1.11–2.65)	0.009
**Marital status**					
Single	85(39.7)	809(42.2)	1	1	
Married	129(60.3)	1107(57.8)	1.11 (0.83–1.48)	1.11 (0.81–1.49)	0.481
**Education**					
No formal education	11(5.1)	198(10.3)	1	1	
Primary	63(29.4)	619(32.3)	1.83 (0.97–3.71)	1.80 (0.95–3.67)	0.074
Secondary or higher	140(65.4)	1099(57.4)	2.29 (1.26–4.52)	2.24 (1.22–4.49)	0.006

Overall, the HIV status was positively associated with maternal age, parity, number of antenatal visits and level of education. There was no significant association between marital status and HIV results.

## Discussion

This study assessed the acceptability of HIV testing and prevalence of HIV among women who come in labour with unknown HIV status. The overall acceptability was high (88.3%) and 10.0% of those tested were HIV positive. Bulterys (2004) reported that 84% of women accepted HIV testing during labour in the USA [7]. Homsy (2006) reported an acceptability rate of 86% in Uganda [9] and Bharucha (2005) reported a lower acceptability rate (40%) in India [8]. These differences in acceptability may be explained by socio-economic and cultural differences as well as differences in perceived risk of HIV infection. Some reasons given by women for refusing HIV testing in our study were fear of stigma, discrimination and rejection. These reasons may vary between different socio-economic and cultural strata.

The overall HIV prevalence in our study was 10.1%. This prevalence is higher than 6.8% among women of reproductive age [11] and 6.0% among pregnant women with known HIV status [13] in Cameroon. Viani (2006) also reported a higher HIV prevalence among women screened during labour (1.12%) than women screened during antenatal care (0.33%) [14]. This suggests that compared to HIV negative women, HIV positive women are less likely have a known HIV status during labour. This highlights the importance of HIV testing during labour and provision of antiretroviral prophylaxis against MTCT of HIV. We used parallel testing for HIV. A systematic review of literature of rapid point-of-care HIV testing in pregnant women found this method of HIV testing to be superior to a single testing strategy with regards to sensitivity and specificity [4].

We reported a negative association between acceptability of HIV testing during labour on one hand, and maternal age and number of antenatal visits on the other hand. This has been reported previously [7]. Some women who had antenatal care with unknown HIV status had previously refused HIV testing and were therefore less likely to accept HIV testing compared to women who were counseled for the first time. Educated women were more likely to accept HIV counseling and testing than women who had no formal education. Educated and uneducated women may differ in their perceived risk of HIV and understanding of the importance HIV testing. For example in one study, educated women were less likely to believe that HIV truly exists, compared to their uneducated counterparts [15]. Wealth and education have been reported to be positively correlated with unsafe sexual behaviour in Cameroon [16]

Voluntary HIV counseling and testing in the delivery room is a challenging task especially in resource-constraint settings because insufficient number of trained staff, and inadequate resources [17]. There is need to have round-the-clock services including laboratory technicians, trained counsellors and access to HIV test kits. Despite these challenges, HIV counseling and testing in delivery room is feasible in both low, medium and high-income countries [7,8]. Wright (2000) suggested that the decision to implement HIV testing in delivery room should depend on the HIV prevalence, access to health facilities, availability of resources, access to delivery skilled health personnel, and cooperation of hospital staff and administrators [18]. The cost of rapid HIV testing in our study was 4 UD$ per person tested.

Other internationally recommended regimens for PMTCT of HIV have superseded Nevirapine alone. World Health Organisation currently recommends two types of regimens [19]: (a) Antiretroviral Therapy if CD4 count is < 200/mm^3 ^irrespective of the WHO Clinical Stage or CD4 count < 350/mm^3 ^plus Clinical Stage 3 or 4 – Zidovudine (AZT) + Lamivudine (3TC) + Nevirapine (NVP) twice daily to women during antepartum, intrapartum and postpartum period and AZT for 7 days to infant; (b) Antiretroviral Prophylaxis where women are given AZT after 28 weeks of pregnancy or as soon as possible thereafter, NVP + AZT/3TC during labour, AZT/3TC for 7 days postpartum and infants are given NVP + AZT for 7 days.

All the antiretroviral drugs recommended by the WHO for prevention of mother-to-child transmission of HIV are available for free to all pregnant women in the four hospitals included in this study. Pregnant women with positive HIV and their infants are put on the appropriate treatment. Therefore intrapartum HIV testing in Cameroon will reduce the number of women who give birth with unknown HIV status and the number of mother-infant pairs who receive treatment for preventing mother-to-child transmission of HIV.

All the four hospitals included in our study are located in the Centre Province in Cameroon and therefore do not accurately represent the whole country. There is need for future research to assess the acceptability of intrapartum HIV testing and counselling in rural areas in Cameroon in order to get a complete picture in the country. In addition, this study did not evaluate the cost-effectiveness of intrapartum HIV counselling and testing. Before the approach is implemented nationwide, it will be important to evaluate the cost-effective of this approach.

## Conclusion

We conclude that HIV prevalence among in labour with unknown HIV status is high, and fortunately enough acceptability for HIV testing during labour is also high. We recommend an opt-out approach for HIV testing during labour in Cameroon (i.e. women are informed that HIV testing will be routine during labour if HIV status is unknown but each person may decline to be tested). An opt-out approach will decrease the proportion of women who give birth with unknown HIV status and increase the number of mother-infant pairs who receive appropriate treatment for preventing mother-to-child transmission of HIV. Bharucha (2004) suggested postpartum counseling and testing of women who refused HIV testing during labour [8]. During the postpartum period women might be better physically and mentally to understand and accept counseling and testing. It might be worthwhile to consider postpartum counseling and testing in settings where HIV prevalence is high. Hospitals with over 10,000 deliveries per year might consider recruiting extra staff to conduct counselling and HIV testing if the maternity staff are faced with high volume of work.

## Competing interests

The authors declare that they have no competing interests.

## Authors' contributions

*EJK *and *ERM *conceived and designed the study, drafted the protocol, analysed data, interpreted the results and wrote all versions of the manuscript. *FXMK, NF, PNN, PMT, RNT and RJIL *critically reviewed the manuscript for important intellectual content. All authors read and approved the final manuscript.

## Pre-publication history

The pre-publication history for this paper can be accessed here:


